# INTERVENTION EFFECTS ON TARGET PROBLEMS EXPRESSED BY CAREGIVERS OF HOME-DWELLING PERSONS WITH DEMENTIA

**DOI:** 10.1093/geroni/igz038.2029

**Published:** 2019-11-08

**Authors:** Richard H Fortinsky, Laura N Gitlin, catherine V Piersol

**Affiliations:** 1 University of Connecticut School of Medicine, Farmington, Connecticut, United States; 2 Drexel University, Philadelphia, Pennsylvania, United States; 3 Thomas Jefferson university, Philadelphia, Pennsylvania, United States

## Abstract

Care of Persons with Dementia in their Environments (COPE) is an evidence-based in-home intervention designed to optimize function and activity engagement in persons with dementia (PWD), and teach family caregivers (CG) how to manage dementia care-related problems. In this presentation, we report problems expressed by CGs, and intervention effects on these problems and CG outcomes, in the COPE CT translational study. CGs randomized to COPE who completed the assessment phase (N=134) expressed a total of 409 target problems, grouped as managing PWD behavioral problems (32%), caring for themselves (30%), managing PWD daily activities (24%) and engaging PWD in meaningful activities (14%). Most problems were reduced (75%) or eliminated (21%) among CG completing the intervention. In preliminary outcome analyses, compared to CG not receiving COPE, CG receiving COPE were more likely to report improved ability to manage dementia-related cognitive and behavioral symptoms (p<0.001). Implications for scaling COPE will be discussed.

